# Crossover point and maximal fat oxidation training effects on blood lipid metabolism in young overweight women: a pilot study

**DOI:** 10.3389/fphys.2023.1190109

**Published:** 2023-06-16

**Authors:** Dizhi Wang, Peizhen Zhang, Jin Li

**Affiliations:** ^1^ School of Sports Medicine and Rehabilitation, Beijing Sport University, Beijing, China; ^2^ Division of Sports Science and Physical Education, Tsinghua University, Beijing, China

**Keywords:** crossover point intensity, maximal fat oxidation intensity, lipid metabolism, overweight, cardiovascular health

## Abstract

**Purpose:** To determine the effects of weight reduction schemes using the exercise intensities corresponding to maximal fat oxidation (FATmax) and crossover point (COP). The effects of different intervention protocols on blood lipid metabolism were compared to explore how fat can be consumed and used more efficiently and provide a theoretical basis for weight loss through exercise.

**Methods:** This study included 30 young overweight women randomly divided into the COP, FATmax, and control groups. Participants in the COP and FATmax groups exercised for 45 min four times a week for 8 weeks after the individual treadmill exercise test. The control group did not perform any exercise.

**Results:** After 8 weeks of training, participants in the COP group significantly decreased weight (2.6 ± 3.3 kg), body mass index (0.91 ± 1.26 kg/m^2^), body fat percentage (1.21% ± 1.50%), and fat mass (1.90 ± 2.30 kg) (*p* < 0.05). They also had significantly decreased hip circumference (4.8 ± 3.3 cm), serum apolipoprotein B (ApoB) levels (15.48 ± 14.19 mg/dL), and ApoB/apolipoprotein AI (ApoAI) ratios (0.23 ± 0.17) (*p* < 0.01). However, their serum ApoAI levels were significantly increased (14.18 ± 10.24 mg/dL; *p* < 0.01). Participants in the FATmax group had significantly decreased hip circumference (2.4 ± 2.0 cm), serum ApoB levels (14.49 ± 11.00 mg/ dL), and ApoB/ApoAI ratios (0.35 ± 0.15) (*p* < 0.01) but significantly increased serum ApoAI levels (29.53 ± 13.29 mg/dL; *p* < 0.01). No significant changes in physiological indexes were observed in participants in the control group.

**Conclusion:** Personalised exercise intervention positively affected central obesity, effectively improving blood lipid metabolism and fat oxidation, reducing cardiovascular disease risk in young overweight women. COP training improved weight and body composition better than the FATmax exercise, while the latter provided greater improvements in serum ApoAI levels.

## 1 Introduction

Age-standardised incidence of weight problems increased from 3.2% in 1975 to 10.8% in 2014 in men and from 6.4% to 14.9% in women. Should those trends persist, the global weight problem incidence will have reached 18% in men and surpassed 21% in women by 2025 (NCD Risk Factor Collaboration, 2016). The World Health Organization reported that the Chinese obese population over 18 years old accounted for 6% of the adult male population, 7% of the adult female population, and 7% of the total adult population. The China Health and Nutrition Survey reported that dyslipidemia prevalence among adults aged 18–59 in China was 37.5% (Huang, et al., 2019). Dyslipidemia has become one of the public health problems endangering health.

Visceral fat accumulation and dyslipidemia are pre-symptoms of obesity-induced diseases (Koster, et al., 2015). Dyslipidemia has an important role in the pathogenesis of various diseases, including coronary heart disease, atherosclerosis, metabolic syndrome, type 2 diabetes, and sarcopenia (Green, et al., 2004). The pathological mechanisms of dyslipidemia caused by obesity are multifactorial. They include excessive secretion of very low-density lipoproteins (VLDL) by the liver, decreased circulating total cholesterol lipolysis, the increased fatty acid flux of fat cells to the liver and other tissues, and small dense low-density lipoprotein (LDL) (Klop, et al., 2013). Many studies have shown that drug treatment and lifestyle changes can improve dyslipidemia and maintain body health (Garelnabi, et al., 2014; Ridker, 2014). Alterations in blood lipid levels caused by lifestyle changes have similar effects to antihyperlipidemic drugs but are safer and more acceptable.

In the process of fat loss during exercise, exercise intensity is a crucial factor affecting the fat oxidation rate. When the exercise intensity increases from medium to high, the glycolysis system becomes the human body’s primary energy supply system. Consequently, lactic acid produced by glycolysis will inhibit long-chain fatty acids from entering the mitochondria, inhibiting fat utilisation (Purdom, et al., 2018). Meanwhile, even consuming the same calories during exercise, the excess post-exercise oxygen consumption differs based on the type and intensity of the exercise. Therefore, the total amount of fat consumed during exercise varies (Jung, et al., 2019).

New training protocols have been designed based on physiological principles, including maximal fat oxidation (FATmax) intensity and crossover point (COP) training. FATmax is the exercise intensity providing maximal fat oxidation (Jeukendrup, and Achten, 2001; Dandanell, et al., 2017). COP is the exercise intensity at which energy from carbohydrate (CHO)-derived fuels surpasses energy from lipids (Brooks, and Mercier, 1994; Purdom, et al., 2018), and studies on COP training are limited (Borel, et al., 2015). Different exercise protocols affect the human body’s physiological mechanisms differently, reflected explicitly in metabolic adaptation (substrate utilisation level), blood lipid improvement effects, and aerobic capacity.

To our knowledge, no previous study has compared the intervention effects of the FATmax and COP exercises. Therefore, it remains unknown which approach best improves the lipid metabolism of young overweight women, and few studies analyse their impact on ApoB/ApoAI ratios. Mendelson et al. (2012) proposed to measure the human body’s COP by running, while other studies have mainly used the bicycle ergometer (Coquart, et al., 2017). There is no well-established COP exercise intervention program based on running, and few studies have compared COP exercise with other exercises. Therefore, this study designed and improved the testing and training scheme for COP exercise under running conditions. In addition, it compared the intervention effects of COP and FATmax exercises on blood lipid metabolism to explore how to consume and utilise fats most efficiently.

## 2 Methods

### 2.1 Participants

Young overweight women were recruited via the internet and flyers and were screened using the physical activity readiness questionnaire and body mass index (BMI). Participants who did not meet the criteria were excluded based on their health status. The screening criteria included: 1) stable weight in the last 3 months; 2) irregular exercise within 1 year; 3) no exercise contraindications, organic diseases, cardiovascular disease, lung disease, hypertension, diabetes, or osteoarticular diseases; 4) no smoking or drinking habits or long-term medication; 5) 24 kg/m^2^ ≤ BMI < 28 kg/m^2^; 6) aged 18–30 years.

Thirty-nine eligible young overweight women were enrolled. After communicating the experiment content and requirement, participants signed the informed consent document. The study protocol was reviewed and approved by the Institutional Review Board of Beijing Sport University (Beijing, China). After the baseline test, participants were divided into the COP, FATmax, and control groups based on their BMI levels. There were 13 women in each group.

Participants took part in the study voluntarily and were told not to take any medicines that might have an impact on the experiment. Before and after the study, the participants’ dietary status was recorded through the diet log, and their physical activity level was evaluated using the short format of the international physical activity questionnaire. Nine participants quit the study due to accidental injuries, to care for family members, or to move to another city. Therefore, 30 women participated in this study: thirteen in the COP group, nine in the FATmax group, and eight in the control group.

### 2.2 Study design

**FIGURE 1:**
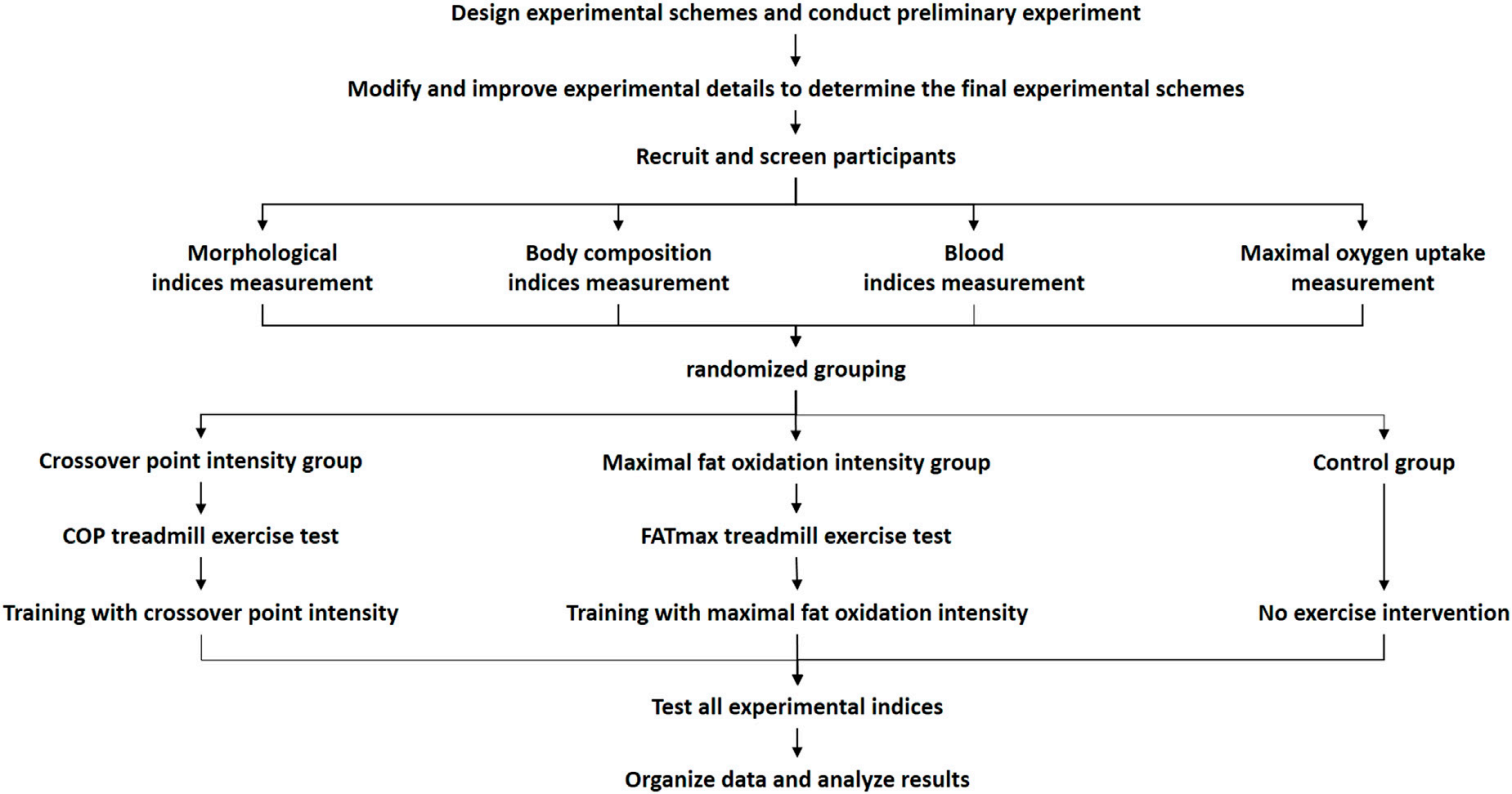
Experimental flow chart.

The preliminary experiments were conducted after designing the overall experimental scheme. Then, based on the preliminary experimental results, the experimental details were modified and improved to develop the final scheme. First, participants were recruited and screened. Second, all participants completed the baseline test, including morphological index measurements, body composition index measurements, blood index measurements, and maximal oxygen uptake measurements. Then, they performed the individual treadmill exercise test. Participants in the COP group performed the COP exercise test, and participants in the FATmax group performed the FATmax exercise test. The COP and FATmax groups completed 45 min of aerobic exercise training four times a week for 8 weeks at heart rates corresponding to COP intensity and FATmax. The exercise training consisted of walking or running on a treadmill. Heart rate monitors (Polar H7) were used to help participants control their target heart rates during exercise. There was a warm-up period of 5 minutes before and a cool-down period of 10 minutes after the exercise. The exercise was supervised by researchers four times per week during the study. Participants in the control group maintained their regular lifestyles during this period. After the 8-week study, various indexes were measured, recorded, and analysed (Figure 1).

### 2.3 Baseline tests

#### 2.3.1 Morphological measurements

Fasting-state morphological indices of participants were measured in the morning. Body weight and height were measured using a standard electronic stadiometer and scale, with light clothing and without shoes. Waist and hip circumference were measured with a medical tape measure according to the standard method. Then, BMI and waist-hip ratio were calculated.

#### 2.3.2 Body composition measurements

Dual-energy X-ray absorptiometry (GE Healthcare Lunar, United States) was used to measure participants’ body composition, including their body fat percentage, lean body mass, abdominal fat, and body fat mass. Participants wore light clothing, took off their shoes and coat, and removed metal items (e.g., keys) and accessories (e.g., earrings and bracelets) before the test. They lay flat on the test bed, eyes closed, and straps fixed the knee and ankle joints.

#### 2.3.3 Blood index measurements

Blood was taken during the follicular phase of the female menstrual cycle (within 12 days of the end of menstruation). Participants were asked to avoid alcohol, coffee, tea, and strenuous exercise 1 day before blood collection. Ten millilitres of venous blood was taken after fasting for 12 h. Blood parameters were analyzed at a certified laboratory using the standard methods and quality control procedures. Serum total cholesterol (TC) and triglyceride (TG) were measured using the oxidase method (Sekisui Medical CO., LTD., Japan). High-density lipoprotein cholesterol (HDL-C) and LDL cholesterol (LDL-C) levels were quantified using the selectively sheltering method (Sekisui Medical CO., LTD., Japan). Apolipoprotein AI (ApoAI) and B (ApoB) levels were quantified using the immune turbidimetry method (Beijing Kangda Taike Medical Technology Co., Ltd., China). Lipoprotein lipase (LPL) levels were determined using an enzyme-linked immunosorbent assay (Human LPL ELISA Kit, Beijing Dongfang Tuojin Technology Co., Ltd., China). All blood samples were processed on the day of collection.

#### 2.3.4 Maximal oxygen uptake measurements

The German Cortex Meta Max 3B portable gas metabolism analyser was used to monitor the ratio of oxygen uptake (VO_2_) and carbon dioxide exhalation (VCO_2_) during exercise and recorded energy metabolism during exercise. The participants wore a portable gas metabolism analyser and performed a maximal oxygen uptake test on a treadmill (h/p/cosmos, Germany). The data changes and the maximal oxygen uptake (VO_2_ max, mL/kg/min) during the exercise were recorded. The Bruce protocol was used as a test solution.

During the test, a 12-lead ambulatory electrocardiograph (AT-10) and an ambulatory blood pressure monitor (Tango+) were used to monitor the dynamic changes in indices. The dynamic electrocardiogram and blood pressure changes were monitored to ensure the safety of the participants. Heart rate was recorded every minute, while blood pressure and rating of perceived exertion (RPE) were recorded every 3 min.

Termination test criteria: 1) Symptoms or signs such as dyspnea, dizziness, tinnitus, nausea, chest pain, extreme fatigue, pale face, or body shaking; 2) Abnormal blood pressure or a decrease in systolic blood pressure between consecutive stages; 3) Systolic blood pressure during exercise ≥250 mmHg; 4) Development of significant electrocardiographic abnormalities, including ST-segment depression ≥2 mm; 5) RPE ≥19; 6) Request to stop to test (Morshedi-Meibodi, et al., 2002).

The maximal metabolic equivalent of energy (MET) value, corresponding to the participant’s maximal oxygen uptake intensity, was calculated using the MET calculation formula (Pescatello, et al., 2014).

$$MET=\frac{Speed\left(m/h\right)\times 0.2+Slope\left(\%\right)\times Speed\left(m/h\right)\times 0.9+3.5}{3.5}$$

### 2.4 COP measurements

Three days after the maximal oxygen uptake test, participants in the COP group wore the gas metabolism analyser and performed the COP exercise test in the morning (8–10 a.m.) after fasting for 12 h. The COP quantitative load test used 20%, 30%, 40%, 50%, and 60% of the participant’s maximum MET value. The COP treadmill protocol was: a 3-min warm-up at 20% of the maximal MET value and then a 6-min quantitative load test at 30%, 40%, 50%, and 60% of the maximal MET value (Pérez-Martin, et al., 2001; Michallet, et al., 2008). The COP treadmill protocol is shown in Table 1.

**TABLE 1:** COP treadmill protocol.

Level (%METmax)	Speed (km/h)	Slope (%)	Duration (min)
20	2.1	0	3
30	4.1	0	6
40	5.0	1	6
50	5.8	2	6
60	6.0	4	6

METmax, maximum MET value.

### 2.5 FATmax measurements

Three days after the maximal oxygen uptake test, participants in the FATmax group wore the gas metabolism analyser and performed the FATmax exercise test in the morning (8–10 a.m.) after fasting for 12 h. The FATmax treadmill protocol was: The participant started exercising at a speed of 5 km/h, increased by 1 km/h every 4 min, with the slope at 0%. The test continued until the participant was exhausted or the respiratory quotient reached ≥1.0 (Venables, et al., 2005; Michallet, et al., 2008).

### 2.6 Exercise intensity

At each exercise intensity level, the average VO_2_ and VCO_2_ of the last 2 minutes were used in Frayn’s stoichiometric equation (Frayn, 1983) to calculate CHO and lipid oxidation rates.

$$Carbohydrate\;oxidation\;rate\;\left(g/\min\right)=4.55\times {VCO}_{2}-3.21\times {VO}_{2}$$

$$Lipid\;oxidation\;rate\;\left(g/\min\right)=1.67\times {VO}_{2}-1.67\times {VCO}_{2}$$

**FIGURE 2:**
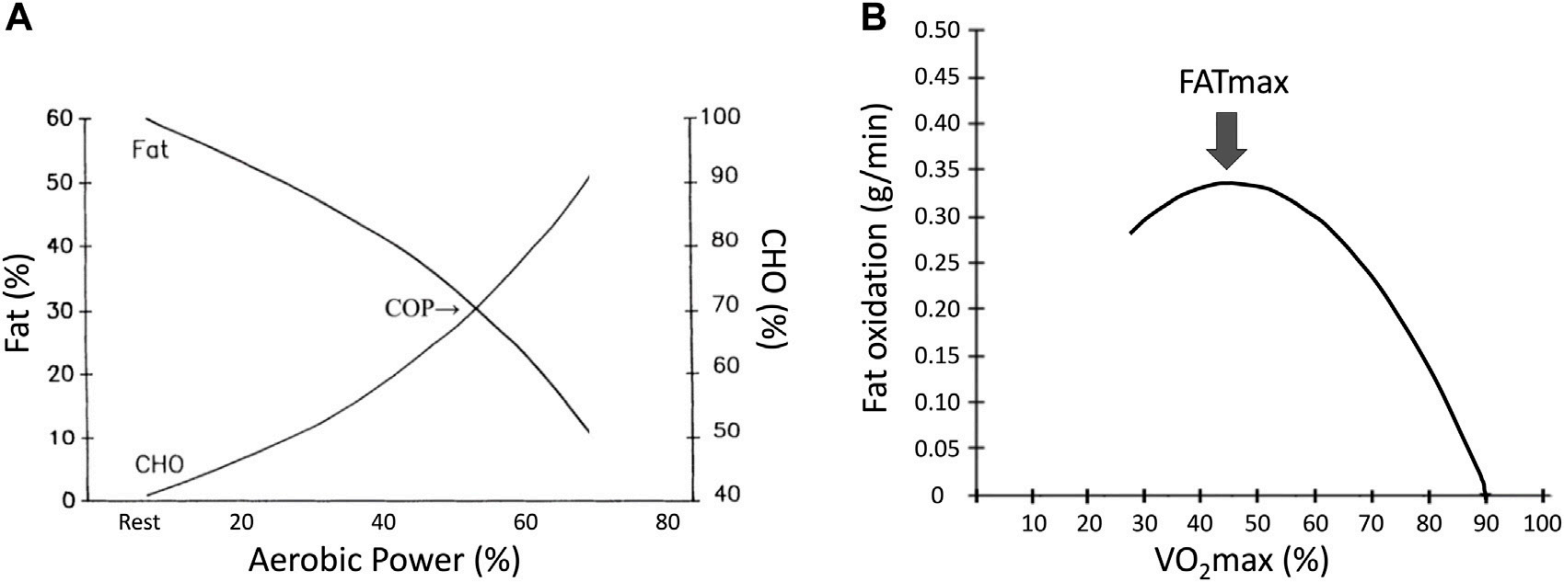
The crossover point and maximal fat oxidation. **(A)**. The crossover point **(B)**. Maximal fat oxidation.

The oxidation rate was used to plot a 2-degree polynomial curve for CHOs and lipids as the intensity increased during exercise. The COP is the exercise intensity at which energy from CHO-derived fuels surpasses energy from lipids, with ∼70% of energy deriving from CHO and 30% from lipids (Pérez-Martin, et al., 2001; Michallet, et al., 2008; Coquart, et al., 2017). With the increase in exercise intensity, the lipid energy supply curve tends to increase before decreasing. The lipid oxidation curve’s apex is the maximal fat oxidation, and the corresponding exercise intensity is the maximal fat oxidation intensity (FATmax; Figure 2) (Jeukendrup, and Achten, 2001; Dandanell, et al., 2017; Purdom, et al., 2018).

### 2.7 Statistical analysis

Statistical software, including SPSS v.23.0 and JASP, were used to perform all statistical data analysis. The Shapiro–Wilk normality test was used to confirm the normality of the data set. Levene’s test was used to evaluate the homogeneity of the data’s variance. Normally distributed indices before and after the experiment were compared using paired-sample t-tests. One-way analysis of variance (ANOVA) was used to compare the three groups before and after the experiment. The Tukey–Kramer test was used as the one-way ANOVA post-hoc test. Data that were non-normally distributed or had uneven variance were compared using the non-parametric Kruskal–Wallis H test. The Mann-Whitney U test was used to perform posthoc tests among groups. A *p* < 0.05 indicated a significant difference, while a *p* < 0.01 indicated a very significant difference. Cohen’s *d* was used to calculate the experimental data’s effect size (ES) and classified as: small ES (0.2 ≤ *d* < 0.5), medium ES (0.5 < *d* ≤ 0.8), and large ES (*d* > 0.8) (Buchan, et al., 2011; Lakens, 2013). Cohen had also provided the η^2^ benchmarks to define small (η^2^ = 0.01), medium (η^2^ = 0.06), and large (η^2^ = 0.14) effects (Lakens, 2013).

## 3 Results

### 3.1 Participants characteristics

The baseline characteristics of the three groups are summarised in Table 2. There were no significant differences in participants’ physiological indices at baseline, indicating that the study cohort was uniform and suitable for scientific data comparison.

**TABLE 2:** Baseline data of participants.

Indices	Group (N)		
	COP (13)	FATmax (9)	Control (8)
Age (yr)	21.5 ± 2.0	22.2 ± 3.4	22.8 ± 0.8
Morphological indices			
Height (cm)	163.4 ± 6.3	164.1 ± 5.8	161.0 ± 6.8
Weight (kg)	70.0 ± 11.2	70.1 ± 6.2	68.7 ± 7.8
Waist circumference (cm)	82.7 ± 9.2	82.9 ± 4.4	82.1 ± 5.2
Hip circumference (cm)	103.7 ± 8.0	103.4 ± 4.4	103.6 ± 9.2
BMI (kg/m^2^)	26.14 ± 3.04	26.12 ± 3.25	26.49 ± 2.17
Waist to hip ratio	0.80 ± 0.04	0.80 ± 0.03	0.79 ± 0.03
Waist-to-height ratio	0.51 ± 0.05	0.51 ± 0.04	0.51 ± 0.03
Body composition indices			
Body fat percentage (%)	38.78 ± 4.48	40.17 ± 3.52	39.42 ± 1.67
Abdominal fat (g)	1906.69 ± 638.92	1968.78 ± 526.37	1935.60 ± 348.01
Fat mass (kg)	27.38 ± 6.29	28.23 ± 4.32	27.08 ± 3.13
Lean body mass (kg)	43.20 ± 6.58	42.34 ± 4.24	42.04 ± 7.85
Blood indices			
TC (mmol/L)	4.70 ± 0.59	4.67 ± 0.45	4.45 ± 0.65
TG (mmol/L)	0.93 ± 0.36	1.25 ± 0.64	0.95 ± 0.55
HDL-C (mmol/L)	1.44 ± 0.31	1.36 ± 0.29	1.36 ± 0.05
LDL-C (mmol/L)	2.71 ± 0.56	2.69 ± 0.26	2.54 ± 0.47
Apo AI (mg/dL)	110.87 ± 16.09	99.40 ± 17.77	108.83 ± 16.48
Apo B (mg/dL)	95.37 ± 16.53	97.68 ± 15.26	82.00 ± 16.82
Apo B/Apo AI	0.89 ± 0.26	1.01 ± 0.24	0.75 ± 0.05
LPL (ng/mL)	1.34 ± 0.20	1.38 ± 0.24	1.43 ± 0.21
Maximal oxygen uptake			
VO_2_max (mL/kg/min)	34.9 ± 6.6	35.2 ± 7.6	35.8 ± 5.2

Data are presented as mean ± SD, unless otherwise indicated. N, the number of participants; BMI, body mass index; TC, total cholesterol; TG, triglyceride; HDL-C, high-density lipoprotein cholesterol; LDL-C, low-density lipoprotein cholesterol; Apo AI, apolipoprotein AI; Apo B, apolipoprotein B; LPL, lipoprotein lipase; VO_2_max, maximal oxygen uptake.

There were no significant differences in morphological indices, body composition indices, blood indices, and maximal oxygen uptake among the three groups at baseline. Participants in the COP group performed the COP quantitative load test. Their oxygen uptake at the COP point was 16.69 ± 5.98 mL/kg/min, their respiratory quotient (RQ) was 0.86 ± 0.03, and their corresponding heart rate was 127.1 ± 23.5 bpm. Participants in the FATmax group performed the maximal fat oxidation exercise test. Their oxygen uptake corresponding to the maximal fat oxidation (MFO) rate was 17.11 ± 4.54 mL/kg/min, their MFO rate was 0.31 ± 0.05 g/min, and their corresponding heart rate was 132.00 ± 18.61 bpm.

### 3.2 Effect of different training on morphological indices

**FIGURE 3:**
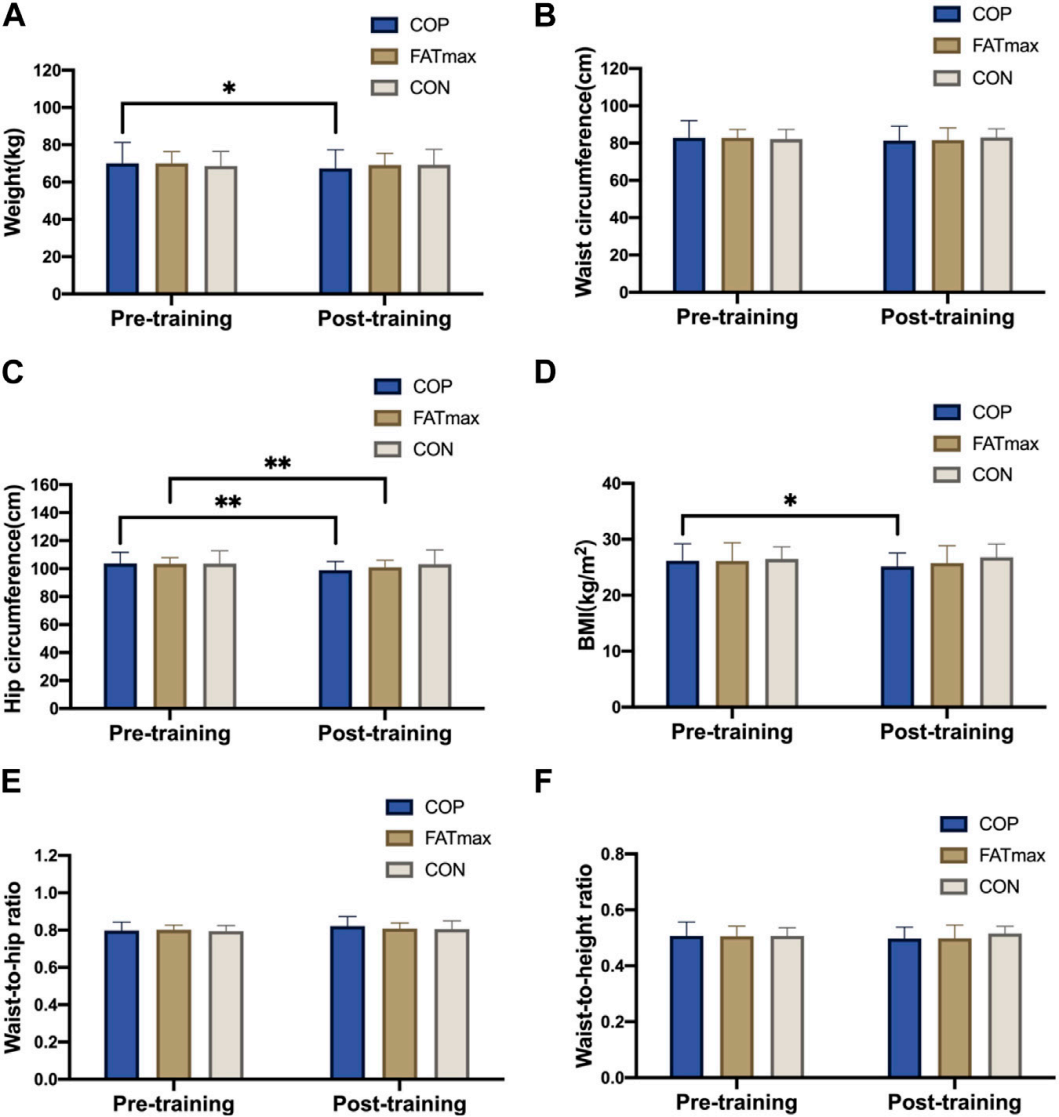
Morphological indices before and after training. BMI, body mass index. Compared with pre-training, * *p* < 0.05, ** *p* < 0.01.

Figure 3 shows changes in morphological indices before and after training. Participants in the COP group had significantly reduced weights (2.6 ± 3.3 kg; 3.71%) after training compared to pre-training (t_(12)_ = 2.94, *p* = 0.012, 95% confidence interval [CI]: 0.69, 4.63; *d* = 0.815, *p* < 0.05). In addition, hip circumference was significantly reduced (4.8 ± 3.3 cm; 4.63%) in the COP group (t_(12)_ = 5.17, *p* < 0.001, 95% CI: 2.75, 6.75; *d* = 1.434, *p* < 0.01). Moreover, BMI was significantly reduced (0.91 ± 1.26 kg/m^2^; 3.48%) in the COP group [(t_(12)_ = 2.94, *p* = 0.012, 95% CI: 0.26, 1.74; d = 0.816, *p* < 0.05)]. Furthermore, hip circumference was significantly reduced (2.4 ± 2.0 cm; 2.32%) in the FATmax group (t_(8)_ = 3.59, *p* = 0.007, 95% CI: 0.88, 4.03; *d* = 1.197, *p* < 0.01). However, no significant within-group changes in morphological indices were observed in the control group. Moreover, no significant differences in morphological indices were observed among groups following training.

### 3.3 Effect of different training on body composition

**FIGURE 4:**
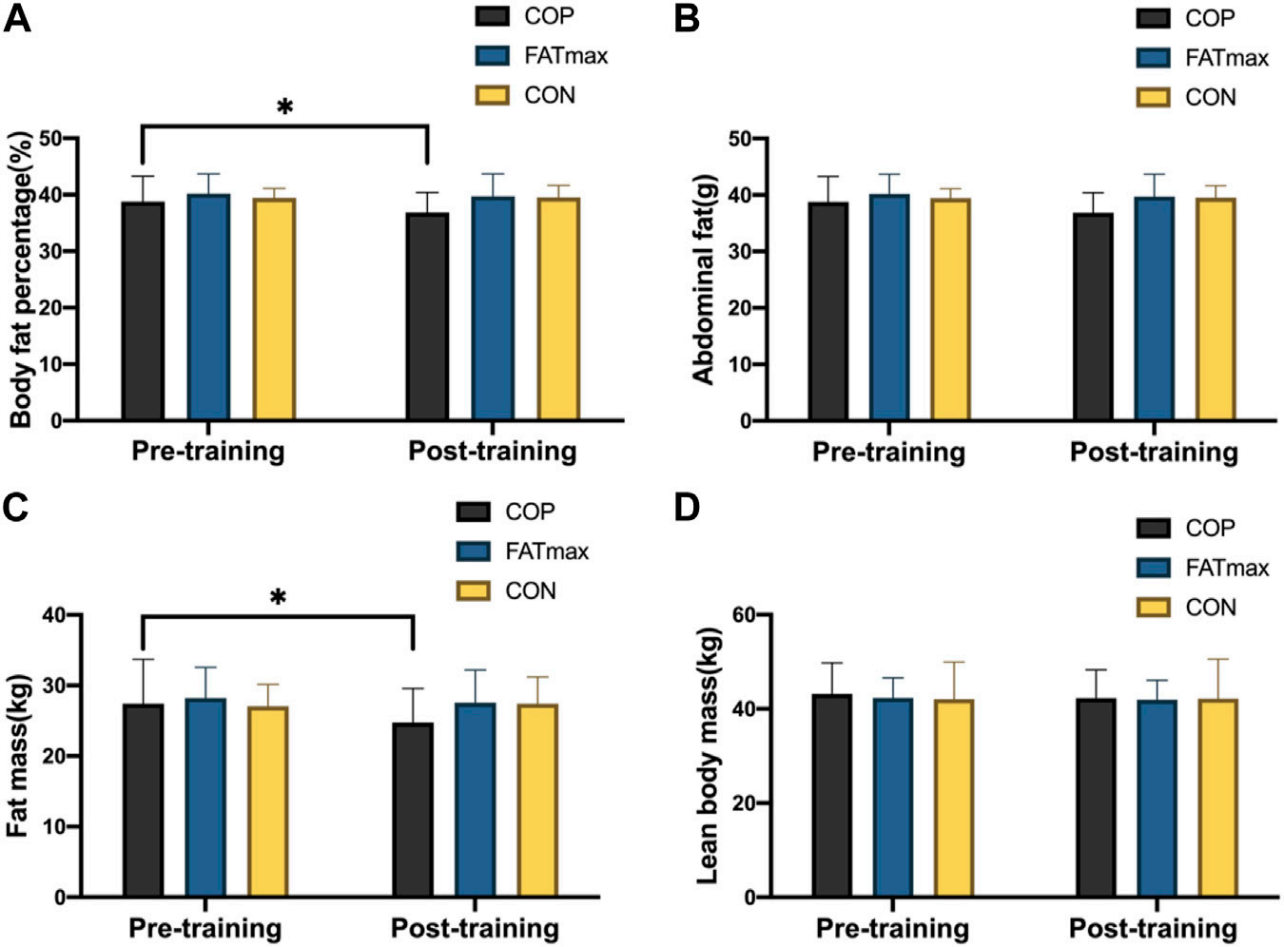
Body composition indices before and after training. Compared with pre-training, * *p* < 0.05, ** *p* < 0.01.

Figure 4 shows changes in body composition indices before and after training. In the COP group, body fat percentage and fat mass decreased significantly from baseline to endpoint (*p* < 0.05). The body fat percentage was significantly reduced (1.21% ± 1.50%; 3.12%) in the COP group [(t_(12)_ = 2.79, *p* = 0.018, 95% CI: 0.25, 2.16; d = 0.804, *p* < 0.05)]. In addition, fat mass was significantly reduced (1.90 ± 2.30 kg; 6.94%) in the COP group [(t_(12)_ = 2.87, *p* = 0.015, 95% CI: 0.44, 3.36; d = 0.827, *p* < 0.05). However, no significant changes in body composition indices were observed in the FATmax and control groups. Moreover, no significant differences in body composition indices were observed among groups following training.

### 3.4 Effect of different training on blood indices

**FIGURE 5:**
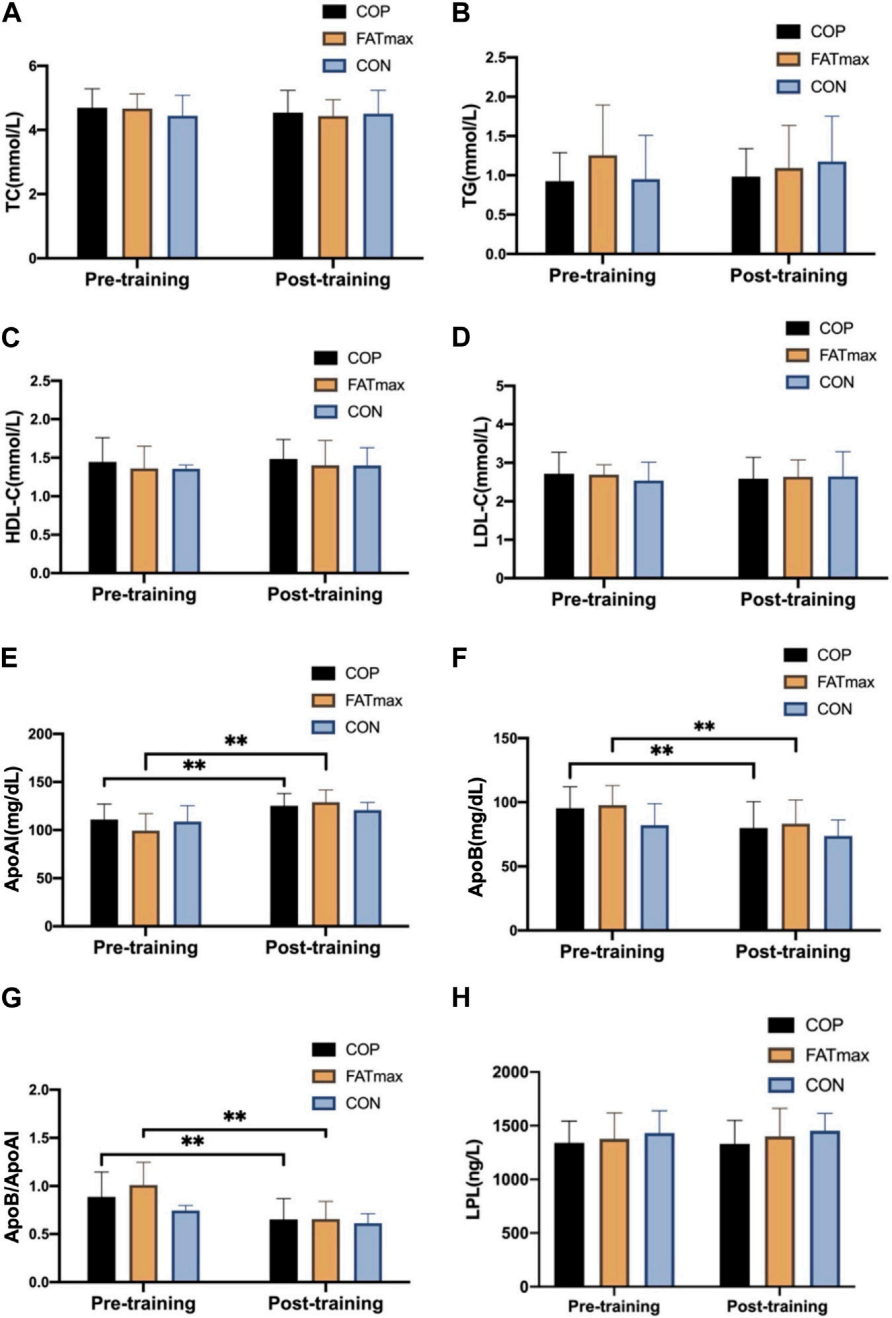
Blood indices before and after training. TC, total cholesterol; TG, triglyceride; HDL-C, high-density lipoprotein cholesterol; LDL-C, low-density lipoprotein cholesterol; Apo AI, apolipoprotein AI; Apo B, apolipoprotein B; LPL, lipoprotein lipase. Compared with pre-training, * *p* < 0.05, ** *p* < 0.01.

Figure 5 shows changes in blood indices before and after training. Compared with pre-training, serum ApoAI concentrations were significantly elevated (14.18 ± 10.24 mg/dL; 12.79%) in the COP group (t_(12)_ = −4.99, *p* < 0.001, 95% CI: −20.36, −8.00; *d* = −1.385, *p* < 0.01). In addition, serum ApoB concentrations were significantly reduced (15.48 ± 14.19 mg/dL; 16.23%) in the COP group (t_(12)_ = 3.94, *p* = 0.002, 95% CI: 6.91, 24.06; *d* = 1.092, *p* < 0.01). Moreover, ApoB/ApoAI ratios were significantly reduced (0.23 ± 0.17; 25.84%) in the COP group [(t_(12)_ = 5.05, *p* < 0.001, 95% CI: 0.13, 0.34; d = 1.401, *p* < 0.01)].

Serum ApoAI concentrations were significantly elevated (29.53 ± 13.29 mg/dL; 29.71%) in the FATmax group (t_(8)_ = −6.67, *p* < 0.001, 95% CI: −39.75, −19.32; *d* = −2.222, *p* < 0.01). In addition, serum ApoB concentrations were significantly reduced (14.49 ± 11.00 mg/dL; 14.83%) in the FATmax group (t_(8)_ = 3.95, *p* = 0.004, 95% CI: 6.04, 22.94; *d* = 1.318, *p* < 0.01). Moreover, ApoB/ApoAI ratios were significantly reduced (0.35 ± 0.15; 34.65%) in the FATmax group [(t_(8)_ = 7.19, *p* < 0.001, 95% CI: 0.24, 0.47; d = 2.397, *p* < 0.01)]. However, no significant changes in blood indices were observed in the control group. Moreover, no significant differences in blood indices were observed among groups following training.

### 3.5 Changes (final–baseline) in indices over time

**FIGURE 6:**
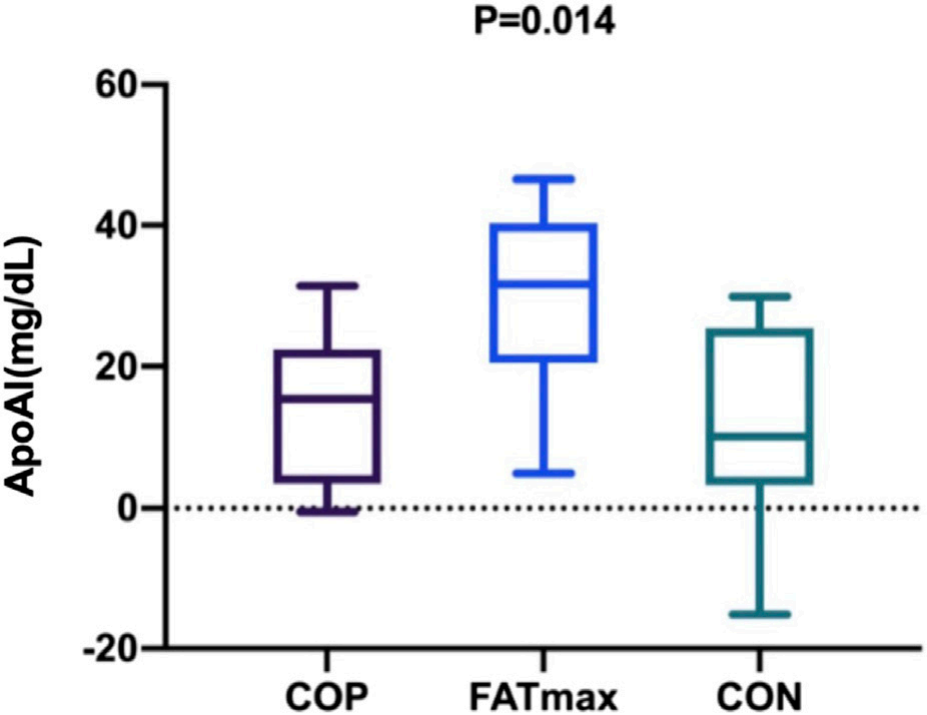
Changes in serum Apo AI (Final–Baseline) over time. Apo AI, apolipoprotein AI.

Figure 6 shows changes in participants’ serum ApoAI concentrations over time. After the personalised exercise intervention, changes in physiological indices over time were not significantly different between exercise groups except for serum ApoAI concentration. There was a significant difference in the changes in serum ApoAI concentrations among participants in the three groups (F_(2,27)_ = 5.396, *p* = 0.014; η^2^_p_ = 0.286; ω^2^_p_ = 0.227; 95% CI: 0.04, 0.45). Tukey–Kramer tests showed that serum ApoAI concentrations in the FATmax group were 15.36 ± 5.42 mg/dL (95% CI: 1.93, 28.79) higher than in the COP group (*p* = 0.023). In addition, serum ApoAI concentrations in the FATmax group were 17.63 ± 6.07 mg/dL (95% CI: 2.58, 32.68) higher than in the control group (*p* = 0.019).

## 4 Discussion

This study designed and evaluated the effect of a COP training protocol and compared the improvements in blood lipid metabolism of young overweight women with two exercise protocols. There are three main findings from this study. First, the physiological indices of young overweight women improved after 8 weeks of COP training. Weight, BMI, hip circumference, body fat percentage, fat mass, serum ApoB concentrations, and ApoB/ApoAI ratios decreased significantly, while serum ApoAI concentrations increased significantly. Second, young overweight women who participated in 8 weeks of FATmax training showed significantly increased serum ApoAI concentrations and significantly decreased hip circumference, serum ApoB concentrations, and ApoB/ApoAI ratios. Third, COP training had a better effect on improving weight and body composition indices. However, FATmax exercise provided benefits similar to COP exercise in blood lipid metabolism. We believe this is the first study to examine and compare the effects of COP and FATmax training on young overweight women’s blood lipid metabolism based on blood lipids, morphology, and body composition.

With the further study of physiological mechanisms in sports, various training methods based on dynamic changes in exercise intensity and human energy consumption have been proposed, including FATmax and COP training. However, it remains unknown which exercise methods bring the greatest health benefits and whether they will differ in their effects on improving blood lipids in young overweight women. These issues warrant further investigation.

In this study, weight and BMI were significantly reduced in the COP group (3.71% and 3.48%, respectively; ES: 0.815 and 0.816; *p* < 0.05), while the weight and BMI were unchanged in the FATmax and control groups. In contrast, Tan et al. (2016) showed that weight and BMI decreased significantly after 8 weeks of FATmax training. The possible reason for this difference is that participants in Tan’s study were middle-aged women, and the exercise time was 300 min per week (1 hour five times a week). In comparison, the FATmax group in this study exercised 180 min per week (45 min four times a week). Therefore, given the differences in total exercise per week, 180 min of FATmax training per week for 8 weeks may be insufficient to improve weight and BMI in young overweight women. However, COP training for the same length of time can effectively promote improvements in weight and BMI, suggesting that COP training is more effective in weight management.

Waist circumference and hip circumference are commonly used indices that comprehensively reflect the participants’ fat distribution status and somatotype. Visceral fat has been associated with an increased risk of death (Koster, et al., 2015). In this study, the hip circumference in the COP and FATmax groups was significantly reduced (4.63% and 2.32%, ES: 1.434 and 1.197; *p* < 0.01). In addition, other body circumference indices showed an improvement trend (*p* > 0.05), suggesting that personalised exercise intervention positively affected central obesity.

In this study, the body fat percentage and fat mass of participants in the COP group were significantly reduced (3.12% and 6.94%, respectively; ES: 0.804 and 0.827; *p* < 0.05). Participants in the FATmax group also had decreased body composition indices. However, body composition indices did not differ significantly in the control group. While the amounts of COP and FATmax training were similar in this study, COP training improved the body composition indices more than FATmax training. This finding suggests that their differing impacts on body composition indices are not due to differences in exercise amount and potentially reflect different metabolic adaptations to different types of exercise.

Studies have shown that endurance training results in muscular biochemical adaptations that enhance lipid oxidation and decrease the sympathetic nervous system response to submaximal exercise stresses. These adaptations promote fat oxidation during mild-to moderate-intensity exercise (Brooks, and Mercier, 1994). The underlying mechanism might be that exercise changes the muscle fibre type, increasing muscle content and free mitochondria density (Bircher, and Knechtle, 2004) and promoting fatty acid translocase (*FAT*/*CD36*) expression (Pelsers, et al., 2008) in skeletal muscle. Exercise-stimulated skeletal muscles secrete exosomes into the cardiovascular system, which promote peripheral and distal organ crosstalk and induce the body’s personalised metabolic adaptability. Exerkines in exosomes included peptides, metabolites, DNA, and various RNA species (Safdar, et al., 2016). Different types of training activate the metabolic response mechanism differently, reflected in the changes in muscle fibre types, mitochondrial volume and density, related protein levels, and exosomes. Therefore, changes in body composition indices after different training also differ.

Multiple epidemiological studies have shown that higher serum TC levels are associated with greater coronary heart disease risk (Kannel, 1983). Increased serum TG levels are associated with an increased ischemic stroke risk (Zivanovic, et al., 2018). HDL-C plays a protective role in atherosclerosis prevention and development (Soška, et al., 2012). LDL-C is a recognised risk factor for coronary heart disease (Chen, et al., 2016). In this study, TC, TG, and LDL-C concentrations in the COP and FATmax groups tended to decrease after 8 weeks of training, while HDL-C concentrations tended to increase. In contrast, the blood lipid indices in the control group were relatively stable. Similarly, Zhu et al. found no significant differences in blood lipid indices of college students after 12 weeks of 90 min/week of aerobic exercise intervention. However, serum HDL levels showed an upward trend, LDL levels showed a downward trend, and a positive development trend appeared (Zhu, et al., 2014), which was consistent with our results in this study.

Recent prospective studies have shown that ApoAI, ApoB, and the ApoB/ApoAI ratio are better predictors of cardiovascular and coronary event risk than conventional clinical lipid measurement indices such as LDL-C, TC, TG, and HDL-C (Song, et al., 2015; Borén, and Williams, 2016; Kim, et al., 2017; Sarzynski, et al., 2018; Zivanovic, et al., 2018). ApoAI is one of the main HDL components and has anti-inflammatory, antioxidant, anti-platelet aggregation, antirejection, antiviral, and anti-angiogenic activities and is beneficial in obesity-related treatment (Zhang, and Zhang, 2017). ApoB is the primary apolipoprotein type of VLDL, intermediate-density lipoprotein, and LDL in the atherosclerotic lipoprotein family and has a similar risk prediction ability to LDL-C (Leiviskä, et al., 2011).

The appropriate range of serum ApoAI concentration in China is 120–160 mg/dL (Yan, 2008). However, in this study, the participants’ serum ApoAI concentrations in the COP, FATmax, and control groups at baseline were all lower than the normal range, suggesting that the participants had some degree of dyslipidemia before training, and their lipid metabolism was impaired. After 8 weeks training, serum ApoAI concentrations increased significantly in the COP (12.79%, ES: −1.385, *p* < 0.01) and FATmax (29.71%; ES = −2.222, *p* < 0.01) groups. In contrast, serum ApoB concentrations decreased significantly in the COP (16.23%; ES: = 1.092, *p* < 0.01) and FATmax (14.83%; ES = 1.318, *p* < 0.01) groups. However, they were unchanged in the control group. Therefore, exercise significantly improved serum lipoprotein levels in participants.

One possible explanation is that aerobic exercise increases the expression of ATP-binding cassette transporter A1 (*ABCA1*) and ApoAI, inhibiting atherosclerosis. The production and reconstruction of plasma HDL-C require various factors, including ATP-binding cassette transporters such as ABCA1, which is a key element of the reverse cholesterol transport pathway. ABCA1 is responsible for the lipidation of lipid-poor ApoAI by cellular cholesterol and phospholipids, a rate-limiting process in both HDL production and cholesterol efflux (Tofighi, et al., 2015). ABCA1 facilitates the delivery of phospholipids from the cell membrane to lipid-poor ApoAI, forming ApoAI from HDL.

Exercise also improves skeletal muscle LPL activity and promotes catabolism of chylomicron and VLDL. Exercise reduces serum ApoB concentrations by promoting VLDL and chylomicron catabolism (Holme, et al., 2007). However, it should be noted that after 8 weeks training, there were significant differences in the changes in serum ApoAI concentrations among the three groups(F_(2,27)_ = 5.396, *p* = 0.014; η^2^_p_ = 0.286, ω^2^_p_ = 0.227; 95% CI: 0.04, 0.45). The possible reason for these differences is that, during exercise, fat is broken down into free fatty acids and glycerin by LPL, transported to muscles through the blood circulation system, and oxidised and broken down by mitochondria in the muscle. Long-term exercise can improve mitochondrial function (Tjønna, et al., 2008) and the activity of enzymes such as LPL and lecithin cholesterol acyltransferase (Klop, et al., 2013; Mann, et al., 2014) and promote the expression of cholesterol ester transfer protein (Mann, et al., 2014). Therefore, exercise caused differences in human physiological parameters such as mitochondrial function, enzyme activity, vascular endothelial cell function, and gene expression, leading to differences in serum ApoAI concentrations in participants.

The ApoB/ApoAI ratio may reflect the balance between potentially atherogenic and anti-atherogenic lipoprotein cholesterol particles. Ben Ounis et al. (2010) showed that 8 weeks of FATmax exercise significantly reduced the ApoB/ApoAI ratios of obese children. Similarly, ApoB/ApoAI ratios decreased significantly in our COP (25.84%, ES = 1.401, *p* < 0.01) and FATmax (34.65%; ES = 2.397, *p* < 0.01) groups, but remained unchanged in our control group. Therefore, personalised exercise can significantly reduce ApoB/ApoAI ratios and disrupt the balance between potential atherosclerosis and anti-atherosclerotic lipoprotein cholesterol particles. Exercise shifted the ratio in a healthier direction, reducing participants’ cardiovascular disease risk.

To our knowledge, this is the first study to examine and compare the effects of COP and FATmax training on blood lipid metabolism in young overweight women based on blood lipids, morphology, and body composition. Both exercises were proposed based on the dynamic physiological substrate metabolism mechanism during exercise. In addition, we have innovatively designed and improved running COP testing and training protocols. Moreover, this study is the first to investigate changes in the blood lipid metabolism of young overweight women after COP training and to compare the effects of two exercise interventions on blood lipid metabolism based on substrate metabolism.

This study had several limitations. First, while participants’ four blood lipid indices did not improve significantly after 8 weeks of training, we did observe significant improvements in their serum lipoprotein and other indices. Future studies should explore the effect of longer training time on blood lipids in young overweight women. Second, we did not conduct a dietary survey to participants. However, the participants were instructed to follow their normal routines and their daily diet was supervised by collecting a diet diary to minimize the impact of diet on the outcomes of current study. Future studies could further explore the effect of dietary intervention combined with COP training on young overweight women. Third, it only explored the effects of different exercise protocols on young overweight women aged 18–30 and did not include male participants. Therefore, it could not determine whether gender and age impact lipid metabolism after personalised exercise intervention. Consequently, our findings may not extend to men and women of other ages.

## 5 Conclusion

An 8-week personalised exercise intervention positively affected central obesity, which can improve the body’s blood lipid metabolism and enhance its ability to oxidise fat. Personalised exercise interventions enhance the body’s positive metabolic adaptability, reducing cardiovascular disease risk in young overweight women. Eight weeks of COP training improved weight, BMI, hip circumference, body fat percentage, and fat mass in young overweight women, while FATmax training only improved hip circumference. However, FATmax training provided similar benefits to COP exercise in blood lipid metabolism. These findings suggest that COP training improves body weight and composition better than FATmax training.

## Data Availability

The raw data supporting the conclusions of this article will be made available on request from the corresponding author. The data are not publicly available due to ethical restrictions.
