# Corrigendum: Crossover point and maximal fat oxidation training effects on blood lipid metabolism in young overweight women: a pilot study

**DOI:** 10.3389/fphys.2024.1426727

**Published:** 2024-07-30

**Authors:** Dizhi Wang, Peizhen Zhang, Jin Li

**Affiliations:** ^1^ School of Sports Medicine and Rehabilitation, Beijing Sport University, Beijing, China; ^2^ Division of Sports Science and Physical Education, Tsinghua University, Beijing, China

**Keywords:** crossover point intensity, maximal fat oxidation intensity, lipid metabolism, overweight, cardiovascular health

In the published article, some numbers were transcribed incorrectly.

**1** A correction has been made to **Abstract**, *Results*. These sentences previously stated: “They also had significantly decreased hip circumference (4.8 ± 3.3 cm), serum apolipoprotein B (ApoB) levels (15.48 ± 14.19 mg/dL), and ApoB/apolipoprotein AI (ApoAI) ratios (0.47 ± 0.37) (*p* < 0.01). However, their serum ApoAI levels were significantly increased (14.18 ± 10.24 mg/dL; *p* < 0.01). Participants in the FATmax group had significantly decreased hip circumference (2.4 ± 2.0 cm), serum ApoB levels (14.49 ± 11.00 mg/dL), and ApoB/ApoAI ratios (0.59 ± 0.30) (*p* < 0.01) but significantly increased serum ApoAI levels (29.53 ± 13.29 mg/dL; *p* < 0.01).” The corrected sentences appear below:

“They also had significantly decreased hip circumference (4.8 ± 3.3 cm), serum apolipoprotein B (ApoB) levels (15.48 ± 14.19 mg/dL), and ApoB/apolipoprotein AI (ApoAI) ratios (0.23 ± 0.17) (*p* < 0.01). However, their serum ApoAI levels were significantly increased (14.18 ± 10.24 mg/dL; *p* < 0.01). Participants in the FATmax group had significantly decreased hip circumference (2.4 ± 2.0 cm), serum ApoB levels (14.49 ± 11.00 mg/ dL), and ApoB/ApoAI ratios (0.35 ± 0.15) (p < 0.01) but significantly increased serum ApoAI levels (29.53 ± 13.29 mg/dL; *p* < 0.01).”

**2** A correction has been made to **3 Result**, *3.2 Effect of different training on morphological indices*, Paragraph 1. This sentence previously stated: “Moreover, BMI was significantly reduced (0.91 ± 1.26 kg/m^2^; 3.44%) in the COP group (t_(12)_ = 2.94, *p* = 0.012, 95% CI: 0.26, 1.74; d = 0.816, *p* < 0.05).” The corrected sentence appears below:

“Moreover, BMI was significantly reduced (0.91 ± 1.26 kg/m^2^; 3.48%) in the COP group [(t_(12)_ = 2.94, *p* = 0.012, 95% CI: 0.26, 1.74; d = 0.816, *p* < 0.05)].”

**3** A correction has been made to **3 Results**, *3.3 Effect of different training on body composition*, Paragraph 1. These sentences previously stated: “The body fat percentage was significantly reduced (1.21% ± 1.50%; 4.93%) in the COP group (t_(12)_ = 2.79, *p* = 0.018, 95% CI: 0.25, 2.16; d = 0.804, *p* < 0.05). In addition, fat mass was significantly reduced (1.90 ± 2.30 kg; 9.75%) in the COP group [(t_(12)_ = 2.87, *p* = 0.015, 95% CI: 0.44, 3.36; d = 0.827, *p* < 0.05)].” The corrected sentences appear below:

“The body fat percentage was significantly reduced (1.21% ± 1.50%; 3.12%) in the COP group [(t_(12)_ = 2.79, *p* = 0.018, 95% CI: 0.25, 2.16; d = 0.804, *p* < 0.05)]. In addition, fat mass was significantly reduced (1.90 ± 2.30 kg; 6.94%) in the COP group [(t_(12)_ = 2.87, *p* = 0.015, 95% CI: 0.44, 3.36; d = 0.827, *p* < 0.05).”

**4** A correction has been made to **3 Result**, *3.4 Effect of different training on blood indices*, Paragraph 1. This sentence previously stated: “Moreover, ApoB/ApoAI ratios were significantly reduced (0.47 ± 0.37; 39.17%) in the COP group [(t_(12)_ = 5.05, *p* < 0.001, 95% CI: 0.13, 0.34; d = 1.401, *p* < 0.01)].” The corrected sentence appears below:

“Moreover, ApoB/ApoAI ratios were significantly reduced (0.23 ± 0.17; 25.84%) in the COP group [(t_(12)_ = 5.05, *p* < 0.001, 95% CI: 0.13, 0.34; d = 1.401, *p* < 0.01)].”

**5** A correction has been made to **3 Result**, *3.4 Effect of different training on blood indices*, Paragraph 2. This sentence previously stated: “Moreover, ApoB/ApoAI ratios were significantly reduced (0.59 ± 0.30; 56.73%) in the FATmax group [(t_(8)_ = 7.19, *p* < 0.001, 95% CI: 0.24, 0.47; d = 2.397, *p* < 0.01)].” The corrected sentence appears below:

“Moreover, ApoB/ApoAI ratios were significantly reduced (0.35 ± 0.15; 34.65%) in the FATmax group [(t_(8)_ = 7.19, *p* < 0.001, 95% CI: 0.24, 0.47; d = 2.397, *p* < 0.01)].”

**6**: A correction has been made to **4 Discussion**, Paragraph 3. This sentence previously stated: “In this study, weight and BMI were significantly reduced in the COP group (3.71% and 3.44%, respectively; ES: 0.815 and 0.816; *p* < 0.05), while the weight and BMI were unchanged in the FATmax and control groups.” The corrected sentence appears below:

“In this study, weight and BMI were significantly reduced in the COP group (3.71% and 3.48%, respectively; ES: 0.815 and 0.816; *p* < 0.05), while the weight and BMI were unchanged in the FATmax and control groups.”

**7** A correction has been made to **4 Discussion**, Paragraph 5. This sentence previously stated: “In this study, the body fat percentage and fat mass of participants in the COP group were significantly reduced (4.93% and 9.75%, respectively; ES: 0.804 and 0.827; *p* < 0.05).” The corrected sentence appears below:

“In this study, the body fat percentage and fat mass of participants in the COP group were significantly reduced (3.12% and 6.94%, respectively; ES: 0.804 and 0.827; *p* < 0.05).”

**8** A correction has been made to **4 Discussion**, Paragraph 12. This sentence previously stated: “Similarly, ApoB/ApoAI ratios decreased significantly in our COP (39.17%, ES = 1.401, *p* < 0.01) and FATmax (56.73%; ES = 2.397, *p* < 0.01) groups, but remained unchanged in our control group.” The corrected sentence appears below:

“Similarly, ApoB/ApoAI ratios decreased significantly in our COP (25.84%, ES = 1.401, p < 0.01) and FATmax (34.65%; ES = 2.397, p < 0.01) groups, but remained unchanged in our control group.”

The authors apologize for these errors and state that this does not change the scientific conclusions of the article in any way. The original article has been updated.

